# Irisin Prevents the Effects of Simulated Microgravity on Bone and Muscle Differentiation Markers

**DOI:** 10.1096/fba.2025-00085

**Published:** 2025-07-10

**Authors:** Lorenzo Sanesi, Roberta Zerlotin, Alessio Campiolo, Angela Oranger, Manuela Dicarlo, Clelia Suriano, Ameneh Ghadiri, Graziana Colaianni, Maria Grano, Sara Tavella, Silvia Colucci

**Affiliations:** ^1^ Department of Clinical and Experimental Medicine University of Foggia Foggia Italy; ^2^ Department of Precision and Regenerative Medicine and Ionian Area University of Bari Bari Italy; ^3^ Cellular Oncology Unit IRCCS Ospedale Policlinico San Martino Genoa Italy; ^4^ Department of Experimental Medicine (DIMES) University of Genoa Genoa Italy; ^5^ Department of Translational Biomedicine and Neuroscience University of Bari Bari Italy

**Keywords:** bone, irisin, microgravity, muscle, myoblasts, osteoblasts, osteocytes, random position machine

## Abstract

Microgravity exposure affects both tissues and cells, and, in this regard, one of the most affected targets is the skeletal muscle system due to the significant loss of bone and muscle mass leading to osteoporosis and sarcopenia, respectively. Several efforts are underway to counteract the effects of microgravity, and recent studies on irisin, a myokine with anabolic effects on the musculoskeletal system, have shown promising results. Due to the practical challenges of conducting experiments in actual microgravity, different devices generating a simulated microgravity condition on Earth have been developed. Here, we exposed myoblasts, osteoblasts, osteocytes to a random position machine (RPM) for five days to assess microgravity effect on the expression of key differentiation factors in cells untreated or treated with irisin. In myoblasts (C2C12), exposure to RPM led to increased expression of early myogenesis maker genes *Pax7* (*p* = 0.0016), *Myf5* (*p* = 0.0005) and *MyoD* (*p* = 0.0009). Irisin treatment in the last 8 h of RPM cultures prevented these increases by returning *Pax7* (*p* = 0.0008) and *MyoD* (*p* = 0.01) to control values, and only partially *Myf5*. In bone cells, exposure to RPM for 5 days showed no effect in osteoblasts (MC3T3) but decreased the expression of *Pdpn* (*p* = 0.0285) and *Dmp‐1* (*p* = 0.0423) genes in osteocytes (MLO‐Y4). Irisin treatment completely prevented the decline in *Pdpn* (*p* = 0.293) and *Dmp‐1* (*p* = 0.0339) levels. Overall, our data showed that the impact of RPM exposure keeps myoblasts and osteocytes in a proliferative state, and irisin treatment restores them to their baseline biological condition, suggesting that irisin can counteract the changes induced by simulated microgravity.

## Graphical abstract

In the RPM condition, irisin treatment reduces the expression of key genes involved in the myogenesis process and increases those of osteocyte differentiation.

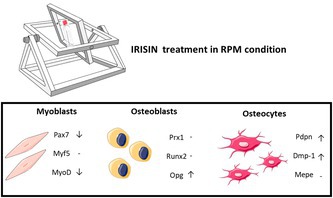

## 1 Introduction

Life on Earth and the human body are subject to the force of gravity, normally unnoticed [1], but its reduction adversely affects various systems and tissues [2], including the musculoskeletal one [3], which loses bone and muscle mass. In the bone tissue, load changes, sensed by osteocytes, the mechanosensory cells and orchestrators of bone remodeling, result in excessive bone resorption by osteoclasts and decreased matrix deposition by osteoblasts. At the muscle level, mass loss is characterized by a reduction in fiber size and a decrease in muscle strength [3, 4]. These conditions characterize two coupled diseases, osteoporosis and sarcopenia, which mainly affect the elderly but also young individuals (sedentary, bedridden, traumatized, or suffering from other diseases) placing them at high risk of fractures and falls. Osteosarcopenia also affects astronauts in Space, who may lose up to 1%–2% bone mass per month during long‐duration missions [5, 6] or experience a 4% to 8% decrease in muscle volume in the first week of space flight [7, 8, 9]. For this reason, many studies have been conducted in space, aboard the International Space Station, and on Earth in simulated microgravity conditions using animal models or devices for in vitro studies, such as the Random Positioning Machine (RPM) or clinostatic rotation [10]. Regarding bone cells, in RPM experiments cytoskeletal alteration was observed, and downregulation of osteoblast differentiation activity and mineralization were demonstrated by the reduction of gene levels coding for Bone Morphogenetic Protein 2 (*Bmp‐2*), matrix proteins such as collagen type I (*Coll‐I*) and osteocalcin (*Ocn*), factors involved in mitochondrial regulation as well as the delay of osteoblast transition to osteocytes [11, 12, 13, 14]. These latter cells also change the expression of secreted molecules controlling remodeling, mineralization, and phosphate homeostasis, by upregulation of pro‐osteoclastogenic cytokine receptor activator of nuclear factor kappa‐Β ligand, *RankL*, Dentin matrix protein1, *Dmp1*, and Fibroblast growth factor 23, *Fgf23*; and by the reduction of gene levels of sclerostin and *Phex* [15, 16]. Regarding simulated microgravity effects on muscle tissue, the loss of mass and strength characterizing muscle atrophy [17, 18] is accompanied by alteration in the myogenesis process. Indeed, by using a clinostat rotation system, it has been proved that human muscle stem/progenitor cells reduce *Pax7* gene levels, an early marker of myogenesis in two weeks of simulated microgravity [19]. In the RPM condition, C2C12 murine myoblasts maintain their proliferation phenotype due to the protein upregulation of MYF5, which also regulates muscle differentiation, and delay the differentiation process for several days [20]. The simulated microgravity‐dependent alteration of myogenesis was recently proved by using a 3D microphysiological system of engineered muscle tissue (EMT), which also demonstrated the reduction of contractile action [21]. Moreover, primary satellite cells, obtained from biopsies of patients undergoing hip arthroplasty, exposed to prolonged simulated microgravity by RPM underwent cell death [22]. Based on these findings and considering that physical activity counteracts bone and muscle loss, the importance of molecules secreted by muscle tissue after exercise has emerged. Myokines exert beneficial effects on multiple organs and tissues in autocrine, paracrine, and endocrine manners [23, 24]. Irisin, an exercise‐mimetic myokine arising from the cleavage of the extracellular domain of its precursor, FNDC5, on the muscle fiber membrane, plays an anabolic role on bone and muscle [25, 26, 27, 28, 29, 30, 31]. Irisin in vivo prevents and counteracts disuse‐induced osteosarcopenia and accelerates fracture healing in mouse models [26, 32]. In a bed‐rest study, a simulated microgravity model on humans on Earth, it was shown that healthy young males after 10 days had low serum levels of irisin and high levels of the bone formation inhibitor, sclerostin, compared to day one. In addition, bedridden subjects with higher irisin levels were protected from muscle atrophy and senescence due to reduced gene levels of ring finger muscle 1 (*MuRF1*) and the cell cycle inhibitor *p21* [33]. In hindlimb unloading mice, an animal model of simulated microgravity, the FNDC5/irisin system is sensitive to load changes because both the precursor in muscle tissue and the myokine in the serum are reduced [27]. In addition, irisin stimulation of C2C12 myoblasts and MLOY‐4 osteocytes increased the expression of genes implicated in their differentiation and protected them from senescence and apoptosis in vitro [27]. Thus, we investigated the effect of irisin stimulation on myoblasts (C2C12), osteoblasts (MC3T3) and osteocytes (MLO‐Y4) cell lines cultured in simulated microgravity conditions by using the Random Positioning Machine (RPM). The Random Positioning Machine (RPM) simulates microgravity by continuously rotating biological samples around two independent axes in a random manner. This constant reorientation prevents cells from sensing a consistent gravity vector, effectively mimicking the conditions of weightlessness.

## 2 Material and Methods

### 2.1 Reagents

MEM alpha (Minimum Essential Medium alpha) and fetal bovine serum (FBS) were produced by Gibco (Gibco; Thermo Fisher, Waltham, MA, USA), penicillin–streptomycin solution and trypsin were from Euroclone (Euroclone S.p.A., Pero, Lombardia, Italia) while L‐glutamine was supplied by Sigma‐Aldrich (Sigma‐Aldrich, St. Louis, MO, USA).

All the cells loaded on the RPM were placed in a medium with α‐MEM with a variable amount of FBS (10% or 2% in the case of C2C12 to induce differentiation) and since the absence of CO_2_ during the microgravity‐simulated exposure, with HEPES (5.96 g/L), NaHCO3 (700 mg/L) and NaCl (300 mg/L) all reagents were supplied by Sigma‐Aldrich (Sigma‐Aldrich, St. Louis, MO, USA).

Irisin (recombinant mouse) was produced by Adipogen (Adipogen International, San Diego, CA, USA) while the plastic material used for cell cultures was provided by SARSTEDT (SARSTEDT AG & Co. KG, Nümbrecht, Germania). The slideflasks used for all experiments were from Thermo Fisher (Thermo Fisher Scientific Inc., Waltham, MA, USA). The RNA extraction was performed by using the RNA mini kit from Qiagen (Qiagen N.V., Hilden, Germany).

### 2.2 Cell Lines

The cell line C2C12 ( *Mus Musculus* ) was purchased by ATCC (ATCC, Manassas, VA, USA) and amplified up to passage P3.

The cell line MC3T3 ( *Mus Musculus* ) was purchased by ATCC (ATCC, Manassas, VA, USA) and amplified up to the passage P4. This cell line was differentiated with ascorbic acid and beta glycerol‐phosphate (Sigma‐Aldrich, St. Louis, MO, USA) at concentrations of 5 μg/mL and 10 mM, respectively.

The MLO‐Y4 cell line ( *Mus musculus* ) was purchased by Kerafast (Kerafast, Boston, MA, USA) at passage number P33, and amplified up to passage P36. This cell type was cultured with α‐MEM with 10% FBS in plates coated with 0.01% rat tail collagen (Sigma‐Aldrich, St. Louis, MO, USA).

### 2.3 The Random Positioning Machine (RPM)

The random positioning machine (RPM) was produced by Dutch Space and connected to a unit that controls its movements, which in turn was connected to a PC that contains the software to set different types of movements related to the simulation of microgravity. In our case, the Random Mode was used in which the plane rotated on two different axes, randomly changing the direction at a speed that oscillated between 30°/s and 60°/s. The RPM was in a dedicated room at 37°C without CO_2_.

### 2.4 Stimulation With Simulated Microgravity and Irisin of C2C12 and MC3T3 Cell Line

Cells were seeded on each slide flask at a density of 10.000 cells/cm^2^ for a total of 5 flasks for condition (*n* = 5). The conditions were ground control condition (Ctrl), RPM, and RPM with irisin.

After 48 h, cells reached the correct confluence, and the medium was changed for C2C12 with MEM alpha‐2% of FBS and for MC3T3 cell line with MEM alpha‐10% FBS with ascorbic acid and beta‐glycerophosphate. The medium was changed every 2 days, and on day 7 of differentiation, the medium was removed, and each flask was filled without bubbles with HEPES MEM alpha and then left for 5 days in the RPM. Before the end of the stimulation, flasks were removed from RPM and stimulated with irisin at the concentration of 100 ng/mL and then maintained for 8 h in this condition.

### 2.5 Stimulation With Simulated Microgravity and Irisin of MLO‐Y4 Cell Line

Cells were seeded in each slide flask at a density of 10.000 cells/cm^2^ for a total of 5 flasks for the condition (*n* = 5). The conditions were the same as in the previous experiment. After 48 h, cells reached the correct confluence, the medium was removed, and each flask was filled without bubbles with HEPES MEM alpha and then left for 5 days in the RPM. Before the end of the stimulation, flasks were removed from RPM and stimulated with irisin at the concentration of 100 ng/mL and then maintained for 8 h in this condition.

### 2.6 RNA Extraction and qPCR

The RNA extraction was performed by using the RNA mini kit from Qiagen by following the company kit instructions. Reverse transcription was performed using iScript Reverse Transcription Supermix (Bio‐Rad, Hercules, CA, USA). The thermal cycler used is MyCycler (Bio‐Rad, Hercules, CA, USA) and the manufacturer's instructions were followed. Quantitative PCR (qPCR) was carried out using SsoFast EvaGreen Supermix (Bio‐Rad, Hercules, CA, USA) on a CFX96 real‐time system (Bio‐Rad, Hercules, CA, USA) for 40 cycles (denaturation, 95°C for 5 s; annealing/extension, 60°C for 10 s) after an initial 30 s step for enzyme activation at 95°C. The primers were designed through Primer‐BLAST (https://www.ncbi.nlm.nih.gov/tools/primer‐blast/).

All primers span an exon–exon junction. The primer sequences were as follows: Gapdh (S‐acaccagtagactccacgaca, AS‐acggcaaattcaacggcacag); Fndc5 (S‐gtgctgatcattgttgtggtccc, AS‐atcatatcttgcggaggag), AS‐attgttgtggtcctcttc; p21 (S‐gcagaataaaaggtgccacagg, AS aaagttccaccgttctcggg); OPG (S‐gaccacctttatacggacag, AS‐ctcacactcacacactcg); p53 (S‐tcttatccgggtggaaggaaa, AS‐ggcgaaaagtctgcctgtctt); Bcl‐2 (S‐ggacttgaagtgccattggt, AS‐ caggctggaaggagaagatg); Bax (S‐agatgaactggacagcaatatgg, AS‐gcaaagtagaagagggcaacc); Runx2 (S‐tcggagaggtaccagatggg, AS‐tgaaactcttgcctcgtccg); Pdpn (S‐cgtcggagggatcttcattg, AS‐agctctttagggcgagaacctt), beta‐2‐Microglobulin (S‐tgctatccagaaaacccctca, AS‐tttcaatgtgaggcgggtgg); Pax7 (S‐agccgagtgctcagaatcaag, AS‐catccagacggttccctttgt); Myf5 (S‐ctgacggcatgcctgaatgta, AS‐caatccaagctggacacgga); Prx1 (S‐ccagagtcaggtgtggttf, AS‐acctgtacggagaggtgt); Dmp1 (S‐agagcaggagccaggagagc, AS‐ccgatgggtttgttgtggtaagc); Mepe (S‐tgctgccctcctcagaaatatc, AS‐gttcggccccagtcactaga); Pdpn (S‐cgtcggagggatcttcattg, AS‐cgtcggagggatcttcattg); Myogenin (S‐cccaacccaggagatcatttg, AS‐cagttgggcatggtttcgtc); MRF‐4 (S‐ctgctaaggaaggaggagcaaa, AS‐acgatggaagaaaggcgctg).

### 2.7 Statistical Analysis

The data was subjected to the Shapiro–Wilk normality test to evaluate the sample distribution. Parameters were expressed as median and interquartile range using GraphPad Prism 9.5 (GraphPad Software, San Diego, CA, USA). For normally distributed values, we performed ANOVA with Tukey's multiple comparison tests, and for non‐normally distributed values, we used the Kruskal–Wallis multiple comparison test. Values were considered statistically significant at *p* < 0.05.

## 3 Results

### 3.1 Effects of Simulated Microgravity and Irisin Stimulation on Gene Expression of Myoblast Differentiation Markers

**FIGURE 1:**
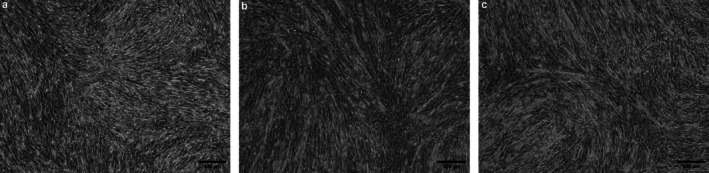
Representative images of myoblasts (C2C12), under different culture conditions. (a) myoblasts cultured in ground control conditions; (b) myoblasts exposed to simulated microgravity for 5 days; (c) myoblasts exposed to simulated microgravity for 5 days and treated with irisin during the last 8 h of RPM exposure (irisin‐RPM).

**FIGURE 2:**
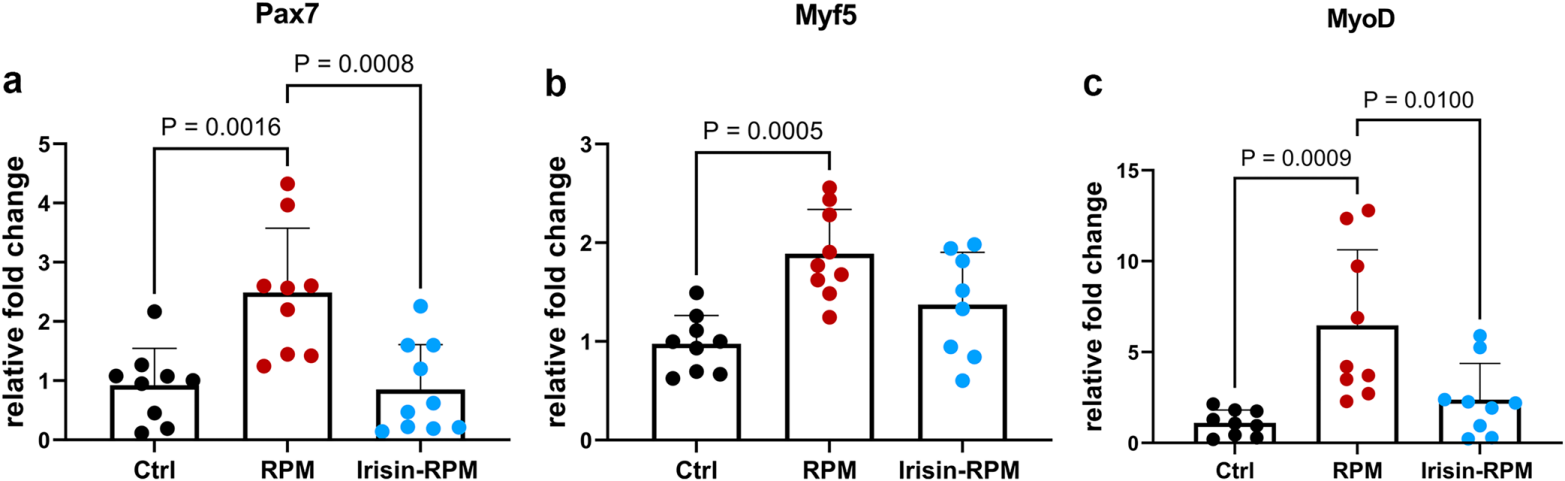
qPCR shows mRNA expression levels of *Pax7*, *Myf5* and *MyoD* in myoblasts (C2C12) cells. Data indicate that irisin treatment prevents the increase of the expression of *Pax7* (a) and *MyoD* (c) and brings the expression to values of control condition, while a partial recovery is observed in *Myf5* (b) expression. Ctrl, control condition; RPM, random position machine.

**FIGURE 3:**
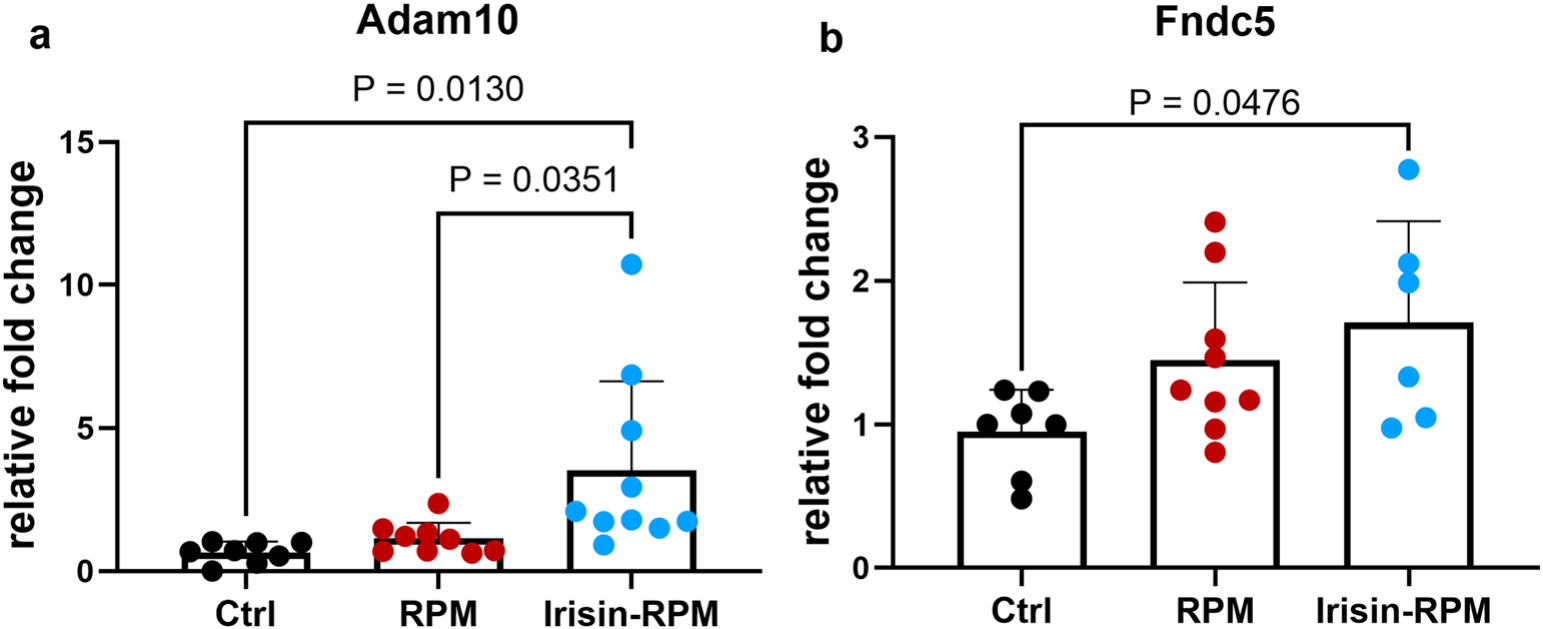
qPCR shows mRNA expression levels of *Adam10* and *Fndc5* in myoblasts (C2C12) cells. (a) irisin treatment increases the gene expression of *Adam10* in RPM condition compared with untreated RPM cell cultures and Ctrl cell cultures. (b) irisin treatment increases the gene expression of *Fndc5* in RPM condition compared with control condition. Ctrl, control condition; RPM, random position machine.

Gene analysis of myogenesis markers was performed on C2C12 myoblasts cultured for 5 days in standard gravity conditions, as control, (Figure 1a, Ctrl) and in RPM conditions with or without the stimulation with 100 ng/mL irisin in the last 8 h (hrs) of RPM cultures (Figure 1b, RPM; 1c, irisin‐RPM). Firstly, it was observed that the morphology of cells in the three different culture conditions was perfectly overlapping and lacking any signs of suffering in RPM with and without irisin compared with the ground control condition (Figure 1). Therefore, on the fifth day of culture, RNA extraction was performed and differences in the gene expression of some regulatory markers of the myogenesis process was detected. RPM conditions in C2C12 myovblasts increased gene levels of *Pax7*, *Myf5*, and *MyoD*, the early regulators of myogenesis, compared to control condition, but when the cells were stimulated with irisin, the expression of the above genes was restored to control levels, suggesting that the presence of irisin in culture reversed the simulated microgravity‐induced gene alteration (Figure 2a–c). No changes in the gene expression of *Myogenin* and *Mrf4*, the late markers of myogenic process, were observed under both simulated microgravity conditions compared with the control condition and in the presence of irisin compared with RPM alone or control condition (data not shown). In addition, simulated microgravity did not induce any change in gene expression of *Adam10* (Figure 3a), the enzyme responsible for cleavage of the irisin precursor, nor of *Fndc5* compared with control, whereas irisin treatment increased the levels of *Adam10* significantly compared with both RPM and control conditions, as well as those of *Fndc5* only compared with control levels (Figure 3a,b).

### 3.2 Effect of Simulated Microgravity and Irisin Stimulation on Gene Expression of Osteoblast and Osteocyte Markers

**FIGURE 4:**
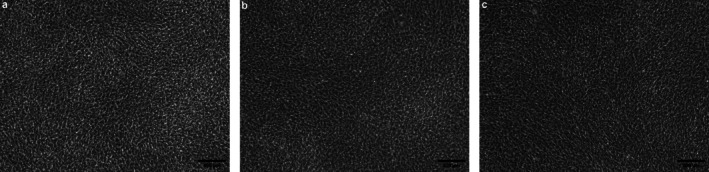
Representative images of osteoblasts (MC3T3), under different culture conditions. (a) osteoblasts cultured in ground control conditions; (b) osteoblasts exposed to simulated microgravity for 5 days; (c) osteoblasts exposed to simulated microgravity for 5 days and treated with irisin during the last 8 h of RPM exposure (irisin‐RPM).

**FIGURE 5:**
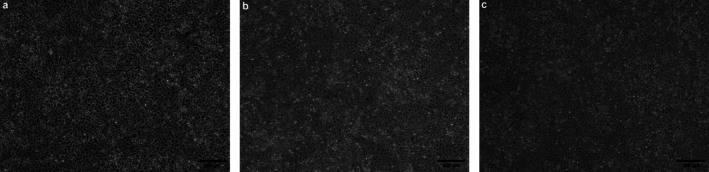
Representative images of osteocytes (MLO‐Y4), under different culture conditions. (a) osteocytes cultured in ground control conditions; (b) ostecytes exposed to simulated microgravity for 5 days; (c) osteocytes exposed to simulated microgravity for 5 days and treated with irisin during the last 8 h of RPM exposure (irisin‐RPM).

**FIGURE 6:**
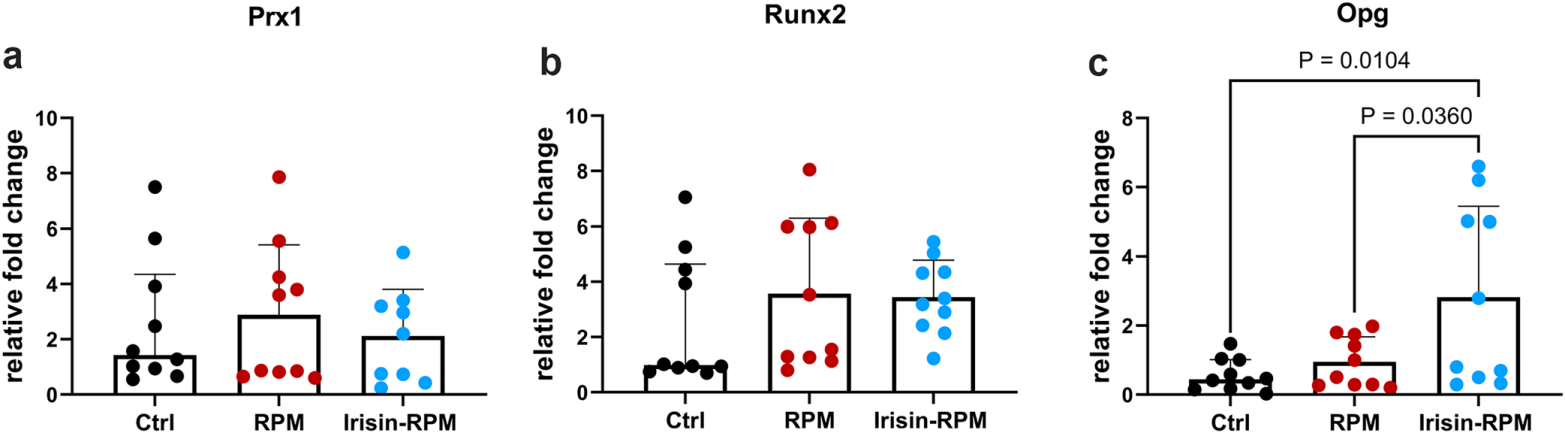
qPCR shows mRNA expression levels of *Prx1*, *Runx2* and *Opg* in osteoblasts (MC3T3) cells. RPM condition does not affect the expression of *Prx1* (a), *Runx2* (b) and *Opg* (c). Irisin treatment of osteoblasts in RPM condition increases gene expression of *Opg* vs. Ctrl condition and RPM condition (c). Ctrl, control condition; RPM, random position machine.

As for myoblasts, gene analysis was carried out for bone cells, particularly murine osteoblast, MC3T3, and osteocyte‐like cells, MLO‐Y4. These cells were cultured for 5 days under standard grounded condition, as control, (Figures 4a, and 5a, Ctrl, respectively) and in RPM with or without the stimulation with 100 ng/mL irisin in the last 8 h of culture (Figures 4b,c, and 5b,c, RPM and irisin‐RPM, respectively). We did not observe changes in overall cell morphology in the three culture conditions. Gene analysis demonstrated that MC3T3 cells in simulated microgravity did not display differences in the levels of *Prx1* and *Runx2* either with or without irisin compared with control conditions. Irisin treatment, instead, upregulated gene expression of *Opg* compared with RPM alone and control levels (Figure 6a–c).

This effect of irisin on OPG modulation was consistent with findings shown in our previous studies, both in space flight experiments with MC3T3 stimulated for 14 days with the same concentration of irisin [34] and in experiments on Earth where the same cells were stimulated in vitro for 8 h with the myokine [27], and also with studies of other authors who stimulated osteoblastic cells with irisin for a longer time (21 days) [35].

**FIGURE 7:**
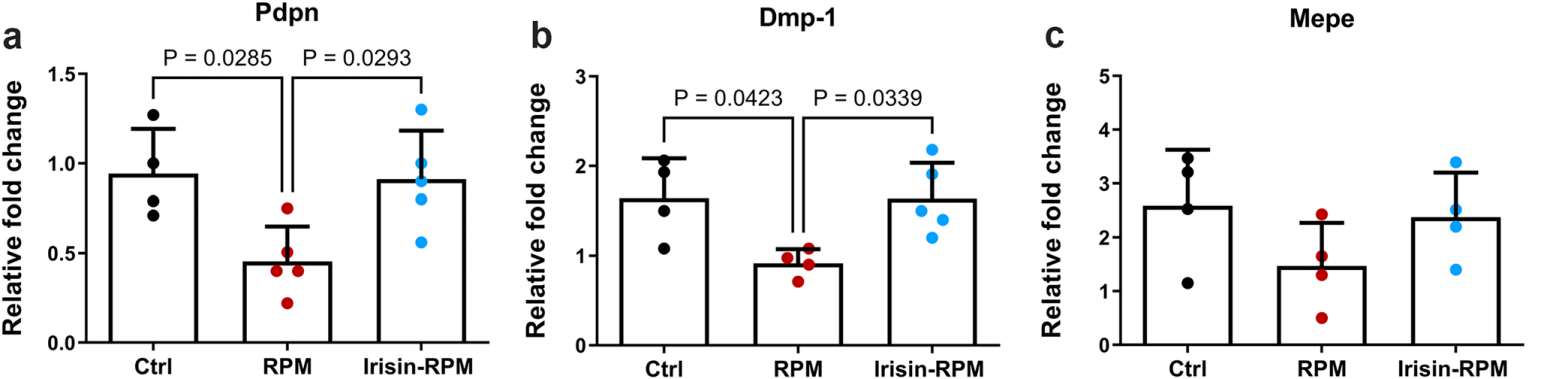
qPCR shows mRNA expression levels of *Pdpn*, *Dmp‐1* and *Mepe* in osteocytes cells (MLO‐Y4). Irisin treatment prevents the decrease in expression observed in RPM conditions for the *Pdpn* (a) and *Dmp‐1* (b) genes and restores gene expression to Ctrl conditions. Exposure to RPM condition does not affect *Mepe* expression (c). Ctrl, control condition; RPM, random position machine.

Gene analysis on MLO‐Y4 showed that under simulated microgravity conditions there were significant decreases in gene levels of podoplanin (*Pdpn*), expressed in the early stages of the differentiation process from osteoblasts into osteocytes, and Dentin Matrix Protein 1 (*Dmp1*), involved in matrix mineralization, as well as a decreasing trend in the levels of Matrix Extracellular Phosphoglycoprotein (*Mepe*), also involved in phosphate homeostasis. MLO‐Y4 stimulation with irisin under RPM conditions showed a significant increase in gene expression of *Pdpn* and *Dmp1* and a trend for *Mepe* compared with the simulated microgravity condition, returning gene levels to those of control (Figure 7a–c).

### 3.3 Effect of Simulated Microgravity and Irisin on Senescence and Apoptosis

**FIGURE 8:**
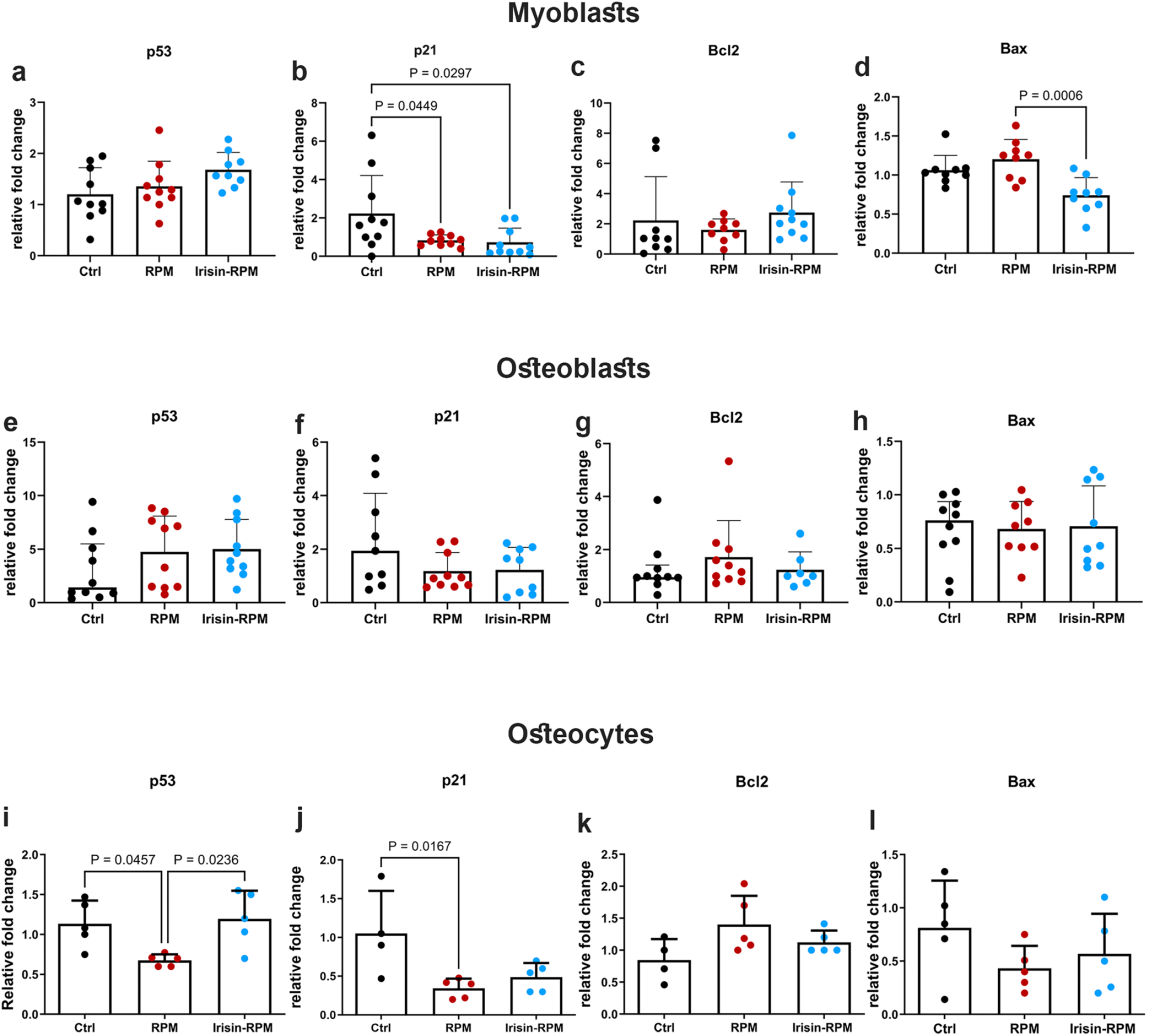
qPCR shows mRNA expression levels of senescence and apoptosis markers in bone and muscle cells. In myoblasts RPM does not alter *p53* (a), *Bcl2* (c) and *Bax* (d) expression, while a decrease of *p21* expression is observed (b). Irisin treatment decreases *Bax* (d) gene expression. In osteoblasts, RPM does not affect gene expression of senescence and apoptosis markers (e,f,g,h). In osteocytes, irisin treatment prevents the drop in *p53* expression (i) observed after RPM exposure and restores values to control values. Moreover, RPM condition decreased *p21* expression both in untreated and irisin group (j). No effect of RPM is observed on *Bcl2* (k) and *Bax* (l) gene expression. Ctrl, control condition; RPM, random position machine.

To better understand the effect of simulated microgravity and irisin stimulation on cellular senescence and apoptosis, we investigated the key factors involved in these processes in myoblasts, osteoblasts, and osteocytes cells cultured as previously described. Investigating *p53* and *p21* senescence gene expression we found that C2C12 myoblasts, in RPM condition with or without irisin stimulation did not show any differences in *p53* levels compared with controls (Figure 8a), while a significant decrease was observed in the levels of *p21 and* the downregulation persisted in the presence of irisin compared with grounded values (Figure 8b). This data suggested that the myokine might contribute to maintaining cells in a proliferating state. The study of gene coding molecules involved in pro‐survival or pro‐apoptotic processes demonstrated that RPM conditions alone did not alter the expression of the anti‐apoptotic *Bcl2* (Figure 8c) or the pro‐apoptotic *Bax*, but the levels of the latter were reduced by the presence of irisin, suggesting its potential protective role against cell apoptosis (Figure 8d).

Regarding bone cells, no significant effects on senescence or apoptosis markers were observed in MC3T3 cells cultured under simulated microgravity conditions either without or with irisin (Figure 8e–h). However, a downregulation of *p53* and *p21* levels was detected in MLO‐Y4 cells compared with grounded values (Figure 8i,j). Irisin treatment completely restored the expression of *p53* to basal levels, while it induced only a partial recovery of p21 expression (Figure 8i,j). Furthermore, no effects on pro‐survival and pro‐apoptotic markers were observed in MLO‐Y4 cells under RPM conditions with or without irisin stimulation compared to controls (Figure 8k,l).

## 4 Discussion

In the present study, we analyzed the effect of simulated microgravity on muscle and bone cells demonstrating that 5 days of RPM changed the levels of some key differentiation genes and irisin treatment generated a regulatory effect by returning their expressions levels to ground control conditions. The effects of microgravity on muscle and bone metabolism have been a broad field of study in recent years [36]. Numerous studies have shown that microgravity has a negative impact on bone metabolism by causing an increase in bone resorption, but this effect has not always been sufficient to explain, for instance, the decrease in bone mass observed after spaceflight [37]. Along with bone, muscle tissue is also affected by microgravity [17], in fact the two tissues are functionally and molecularly linked explaining why osteoporosis and sarcopenia occur together on Earth and in Space.

We previously demonstrated that the myokine irisin plays a critical role in the molecular cross talk between muscle and bone, as it is able to prevent and restore bone and muscle loss in hindlimb suspended mice, a mouse model of disuse‐induced osteosarcopenia [25]. Therefore, we here wondered how simulated microgravity may affect the differentiation process of musculoskeletal cells and whether irisin treatment may influence it. We showed that in myoblasts cultured for 5 days in RPM, *Pax7*, *Myf5*, and *MyoD*, highly expressed in the early stages of myogenesis, were significantly upregulated suggesting that microgravity maintains the cells in a proliferative state. Interestingly, irisin treatment fully prevented the upregulation of *Pax7* and *MyoD*, and only partially prevented that of *Myf5*. These findings are consistent with the study by Calzia et al. [20], which observed that exposure of muscle cells to simulated microgravity induces a state of non‐differentiation, influenced by altered calcium homeostasis.

In addition, the finding that irisin increased gene levels of ADAM10, the well‐known enzyme responsible for cleavage of FNDC5 [38], which is also implicated in the myogenesis process [39], suggests that in myoblasts exposed to RPM, a positive feedback mechanism in the regulation of ADAM10 by irisin might occur.

In this study, we also observed that 5‐day exposure to RPM did not alter FNDC5 levels, but its expression increased in irisin‐treated cells exposed to RPM compared with the baseline biological condition. This latter result is not surprising in myoblasts, as it is in line with data showing that primary myoblasts obtained from mice subjected to free wheel running activity and C2C12 myoblasts treated with irisin in vitro increased FNCD5 expression, as well as muscle fibers of irisin‐treated mice showed higher FNDC5 positivity [26, 40].

However, the ability of irisin to restore the gene expression levels of myogenic factors to those observed under normal gravity conditions suggests a novel role of this myokine in promoting the transition of the myogenesis process from the proliferative phase towards myotube differentiation. RPM‐induced myoblast proliferation is supported by the decrease in *p21* gene expression in both untreated and irisin‐treated C2C12, suggesting that the differentiation delay occurs in cells protected from senescence.

Moreover, exposure to simulated microgravity did not affect the expression of the anti‐apoptotic *Bcl‐2* or the pro‐apoptotic *Bax*. However, irisin treatment decreased the expression of *Bax*, confirming the anti‐apoptotic effect of the myokine, as we previously observed in the cortical bone of hindlimb unloaded mice treated with irisin [27].

In this study, we did not observe any modulation in osteoblasts following RPM exposure. Simulated microgravity did not affect the expression levels of genes associated with cell differentiation, senescence, and apoptosis. These data, which appear in contrast with other studies showing that microgravity decreases osteoblastic differentiation genes in mesenchymal stromal cells (MSCs) and MC‐3 T3 cells differentiated for a short period [41, 42, 43], suggest that these divergent results could be influenced by the differentiative state of the cells. Indeed, in the present work, we studied fully differentiated MC‐3 T3 osteoblasts, cultured in an osteogenic medium for 12 days before undergoing the RPM protocol. In our experiment, mature osteoblasts might have adapted to RPM‐induced microgravity without exhibiting significant changes at the gene expression level. The increase in apoptosis in osteoblasts exposed to simulated microgravity is still debated. The cell death observed following microgravity exposure may explain the reduced bone formation observed during this condition. However, numerous studies have reported that osteoblast apoptosis remained unchanged under simulated microgravity [12, 44]. Furthermore, human bone marrow stromal cells (hBMSCs) cultured aboard the International Space Station (ISS) for 14 days showed reduced expression of osteocalcin and collagen type I, indicating lower differentiation and development, while whole‐genome analysis and next‐generation sequencing indicated no alteration in apoptosis or cellular senescence [10]. Interestingly, we reported that differentiated MC3T3 cells respond to irisin treatment during RPM with an increase in *Opg* expression. The ability of the myokine to upregulate the anti‐osteoclastogenic cytokine was in line with previous research conducted under normal gravity, showing that osteoblasts, through OPG production, can contribute to reduce the excess of bone resorption, a condition occurring also in microgravity [27].

In contrast to osteoblasts, this study found that osteocytes were mainly affected by simulated microgravity. After 5 days of RPM exposure, MLO‐Y4 showed decreased expression of *Pdpn* and *Dmp1* and irisin treatment completely prevented their decrease. In addition, exposure to RPM led to decreased expression of the senescence marker genes *p53* and *p21*, and irisin treatment prevented the drop of p53, restoring it to control values, but was not effective on *p21*. On the other hand, osteocytes are bone cells that sense mechanical stimulation and exposure to microgravity and therefore are the cells most affected by simulated microgravity. Furthermore, a study reported that osteocytes underwent apoptosis as early as three days after exposure to μG during spaceflight, leading to an increase in empty lacunae or lacunae with reduced volume and altered shape [45], accompanied by reduced bone formation and mineralization [45]. Our data suggest that irisin has a strong effect on osteocytes, not only restoring p53 gene expression values, but also promoting their differentiation. These data suggest that it might be a key factor in preventing the bone loss not only observed in microgravity conditions, but also in all those pathologies that lead to bone loss caused by the absence of mechanical loading, as occurs in bedridden patients [31, 33]. Overall, our data revealed the impact of RPM exposure on the expression of key differentiation genes involved in myogenesis and osteocytogenesis processes suggesting the induction of a proliferative state in cells under simulated microgravity conditions. This phenotype can be counteracted by irisin treatment suggesting that irisin is a rebalancing molecule that can restore the grounded state of cells. These results encourage future studies to better understand whether irisin can be used for the prevention and treatment of disuse‐induced musculoskeletal changes in elderly people, astronauts exposed to microgravity during their space missions, as well as in sedentary or bedridden subjects.

## Author Contributions

Lorenzo Sanesi, Roberta Zerlotin, Alessio Campiolo, Angela Oranger, Manuela Dicarlo, Clelia Suriano, Ameneh Ghadiri, Graziana Colaianni, Maria Grano, Sara Tavella, Silvia Colucci: analyzed and interpreted the data. All authors are involved in drafting and revising the manuscript.

## Conflicts of Interest

The authors declare no conflicts of interest.

## Data Availability

Data are available in a publicly accessible repository that does not issue DOIs. Data can be found here: dataset sanesi et al accessed on 17 March 2025.
